# Mitochondrial Dynamic Proteins MiD49 and MiD51 as Novel Targets of Cardioprotection

**DOI:** 10.3390/cells15060559

**Published:** 2026-03-20

**Authors:** Parisa Samangouei, Gustavo E. Crespo-Avilan, Andrew R. Hall, Sauri Hernandez-Resendiz, J. Maeve Elder, Laura D. Osellame, Nicole G. Z. Tee, Khairunnisa Katwadi, Sang-Bing Ong, Xiu-Yi Kwek, Siavash Beikoghli Kalkhoran, Niall Burke, Derek M. Yellon, Derek J. Hausenloy

**Affiliations:** 1 The Hatter Cardiovascular Institute, Institute of Cardiovascular Science, University College London, London WC1E 6HX, UK; 2 National Heart Research Institute Singapore, National Heart Centre, Singapore 16909, Singapore; 3 Cardiovascular and Metabolic Disorders Program, Duke-National University of Singapore Medical School, Singapore 169857, Singapore; 4 La Trobe Institute for Molecular Science, La Trobe University, Melbourne, VIC 3083, Australia; 5 Olivia Newton-John Cancer Research Institute, Heidelberg, VIC 3084, Australia; 6 Yong Loo Lin School of Medicine, National University Singapore, Singapore 117597, Singapore

**Keywords:** ischaemia-reperfusion injury, cardioprotection, mitochondrial dynamics, MiD49, MiD51, mitochondrial fission, mitochondrial fusion, mitochondrial permeability transition pore

## Abstract

Novel therapeutic strategies are required to protect the heart from acute ischaemia-reperfusion injury (IRI) and improve outcomes in patients with acute myocardial infarction (AMI). Mitochondria play a critical role in determining cardiomyocyte fate following acute IRI, with genetic and pharmacological inhibition of Drp1-mediated mitochondrial fission limiting cardiomyocyte death. We investigated the role of the mitochondrial Drp1 receptors, MiD49 and MiD51, as novel targets for cardioprotection. In cardiac cell lines subjected to simulated IRI, dual genetic knockdown of both MiD49 and MiD51 reduced cell death, inhibited mitochondrial fission, prevented mitochondrial permeability transition pore opening, and attenuated mitochondrial calcium overload compared with wild-type cells. However, individual knockdown of either MiD49 or MiD51 did not induce mitochondrial elongation or inhibit MPTP opening. Whole-body genetic ablation of MiD49 in adult mice modestly altered mitochondrial morphology but did not affect myocardial infarct size or cardiac function following AMI. Together with the in vitro protection seen with dual MiD49/51 knockdown, these findings suggest that MiD49 deficiency alone is insufficient and that coordinated inhibition of MiD49 and MiD51 may be required for cardioprotection.

## 1. Introduction

Ischaemic heart disease (IHD) remains the leading cause of morbidity and mortality worldwide, with acute myocardial infarction (AMI) as its most severe manifestation, leading to irreversible cardiac injury following prolonged ischaemia and an increased risk of heart failure (HF) [1,2]. Timely reperfusion is essential to restore blood flow and limit infarct size; however, the abrupt reintroduction of oxygen paradoxically exacerbates cardiomyocyte death through acute ischaemia-reperfusion injury (IRI), which can contribute up to half of the final infarct size [3,4]. Mitochondrial dysfunction is central to IRI pathogenesis, driving excessive reactive oxygen species (ROS) generation, calcium overload, and mitochondrial permeability transition pore (MPTP) opening, hallmarks of IRI-induced cell death [5,6,7].

Mitochondria occupy over one-third of cardiomyocyte volume and supply most of the ATP required for contraction. They are highly dynamic organelles that undergo continuous cycles of fusion and fission to maintain bioenergetic and structural integrity [8,9]. Imbalances in mitochondrial dynamics critically influence cardiomyocyte fate. Mitochondrial fission is mediated by the cytosolic GTPase dynamin-related protein-1 (Drp1). Drp1 is recruited to the outer mitochondrial membrane (OMM) by receptor proteins including MiD49 and MiD51, two highly conserved vertebrate-specific adaptors [10,11,12,13,14]. Although inhibition of Drp1-mediated fission reduces cell death and confers cardioprotection in preclinical models, broad Drp1 inhibition is limited by systemic toxicity and lack of selectivity [10,15,16].

MiD49 and MiD51 have emerged as more selective candidates for modulating mitochondrial fission. Their knockdown prevents Drp1 recruitment, promotes mitochondrial elongation, and reduces apoptosis in multiple cell types [12,17,18]. In the cardiovascular setting, MiD49 downregulation attenuates doxorubicin-induced cardiotoxicity [19], while aberrant expression of MiD49/51 contributes to pulmonary hypertension and mitochondrial myopathy [20,21,22]. Notably, MiD51 expression increases in the heart during IRI, suggesting a role in cardiac stress response [23]. Given the absence of clinically viable mitochondrial-targeted interventions for AMI, a clear understanding of how MiD49 and MiD51 regulate mitochondrial dynamics under conditions of acute IRI could reveal new cardioprotective strategies. In this study, we therefore investigated the role of MiD49 and MiD51 as novel targets for cardioprotection.

## 2. Materials and Methods

### 2.1. Cell Culture

HL-1 cells were cultured as previously described [24]. Cells were maintained in Claycomb medium (51800C, Sigma-Aldrich, St. Louis, MO, USA) supplemented with 10% foetal bovine serum (FBS, F2442, batch 11A568, Sigma-Aldrich), optimised to preserve their cardiac contractile phenotype. Where possible, HL-1 experiments were also repeated using H9c2 cells, as H9c2 cells have been shown to be more energetically similar to primary cardiomyocytes [25]. In certain experimental applied conditions, the increased sensitivity of H9c2 cells precluded adequate model characterisation and resulted in insufficient reproducibility. H9c2 (2-1) were maintained in high-glucose DMEM supplemented with 10% FBS, L-glutamine and penicillin/streptomycin, and were passaged at 70–80% confluence to minimise spontaneous differentiation. H9c2 and HeLa cell lines were sourced commercially. All cell lines were maintained at 37 °C, in a humidified atmosphere containing 5% CO_2_, andCO_2,_ and used between passages 3 and 20 following thawing.

### 2.2. Plasmids

shRNA plasmids targeting MiD49 and MiD51 were kindly provided by Dr. Laura Osellame and Prof. Michael Ryan (University of Monash) [12]. The empty vector (RcCMV), hFis1, Mfn2 (pCB6-MYC-Mfn2), and dominant-negative Drp1^K38A (pcDNA3.1-HA-K38A-DRP1) were obtained from Prof. Luca Scorrano (University of Padua). mitoGCamP6 for mitochondrial Ca^2+^ was kindly provided by Prof. Gyorgy Szabadkai (University College London). Other mitochondria-targeted reporters, including mtRFP, mito-BFP (Addgene #49151), and GFP-Mff (Addgene #49153), were sourced from Addgene (Watertown, MA, USA) or collaborators as noted.

### 2.3. Cell Transfection

Transfection was conducted using X-tremeGENE (Fugene, Roche, Mannheim, Germany) according to manufacturer protocols and optimised for each cell line. Co-transfection of shRNAMiD49/51 and mitochondrial reporters was performed at a 2.5:1 ratio. H9c2 cells were used 48 h post-transfection. HL-1 cells, due to low transfection efficiency, were transfected twice and used 96 h after the initial transfection.

### 2.4. Confocal Microscopy

Fixed and live cells were imaged on a Leica SP5 confocal microscope using 40× and 63× oil-immersion objectives (Leica Microsystems, Mannheim, Germany). Leica Application Suite version 2.6.3.8173 was used for acquisition and analysis. Laser intensity was minimised to prevent phototoxicity.

### 2.5. Mitochondrial Morphology Analysis

Cells were seeded onto 25 mm glass coverslips and fixed 96 h after the first transfection using 4% paraformaldehyde (PFA) pre-warmed to 37 °C for 15–20 min. After washing with PBS, coverslips were mounted onto slides using Dako fluorescence mounting medium (S3023). Confocal images were acquired from randomly selected fields across four or more independent experiments. Mitochondria were visualised using mitochondrial red fluorescent protein. Quantitative scoring was performed by two independent investigators blinded to the treatment groups, using established morphological criteria adapted from classifications used by Palmer et al. 2011 [12]. Mitochondrial morphology was based on the dominant morphology observed (>50%) within each cell, defined as predominantly fused if >50% of mitochondria appeared as elongated rods and/or interconnected networks, or fragmented if <50% mitochondria within the cell appeared as small circular or doughnut-like structures. Analysis from the independent experiments were used to calculate the average percentage of cells showing predominantly elongated mitochondrial morphology in each treatment group.

### 2.6. MPTP Opening Assay

MPTP opening was induced using a laser-triggered oxidative stress protocol as previously described [26,27,28,29]. HL-1 and H9c2 cells were incubated with tetramethylrhodamine methyl ester (TMRM) at 0.5 µM and 1 µM, respectively. Confocal time-lapse imaging was performed every 1.3 s using a scan speed of 400 Hz to monitor changes in TMRM fluorescence. Imaging was continued until a stable fluorescence plateau was achieved. The onset of MPTP opening was defined as the midpoint between peak fluorescence intensity and complete signal loss. Quantification was conducted using Leica Application Suite v2.6.3.8173. A minimum of six independent experiments were conducted for each condition. To maintain TMRM signal integrity and reproducibility, cells were imaged immediately after staining. For validation, cells pre-treated with 0.2 µM cyclosporin A (CsA) were used as positive controls [28,30].

### 2.7. Simulated Ischaemia-Reperfusion Injury (SIRI)

SIRI was modelled to mimic the metabolic and oxidative stress of myocardial infarction as described [31]. The durations of ischaemia and reperfusion were optimised to yield >45% cell death in vector control (VC) cells. Transfected cells were incubated in pre-warmed hypoxic buffer (Na-lactate 10 mM, NaCl 127.8 mM, KCl 14.8 mM, KH_2_PO_4_ 1.2 mM, MgSO_4_ 1.2 mM, CaCl_2_ 1 mM, and NaHCO_3_ 2.2 mM; pH 6.4) [32] and placed in an airtight hypoxic chamber at 37 °C for 7 h. Reperfusion was simulated by replacing the hypoxic buffer with pre-warmed normoxic buffer (D-glucose 10 mM, NaCl 118 mM, KCl 2.6 mM, KH_2_PO_4_ 1.2 mM, MgSO_4_ 1.2 mM, CaCl_2_ 1 mM, and NaHCO_3_ 22 mM; pH 7.4) supplemented with 3 µM propidium iodide (PI) for 1 h at 37 °C [32]. Normoxic control cells were exposed to normoxic buffer for the same duration. As a positive control for cytoprotection, insulin (0.2 µM) was added during the reperfusion phase. Cell death was quantified as the proportion of PI-positive, mtGFP-expressing cells in 10 randomly selected fields per sample (~300 cells/group), across six independent experiments, using a Nikon Eclipse TE200 fluorescence microscope. Image analysis was performed independently by two investigators blinded to group allocation.

### 2.8. Real-Time SIRI Imaging

Real-time imaging of mitochondrial responses during hypoxia and reoxygenation was performed using a Warner PM-2 heated perfusion system mounted on a Leica SP5 confocal microscope. Hypoxic and normoxic buffers [32] were continuously perfused through gas-impermeable tubing while bubbled with appropriate gas mixtures (95% N_2_/5% CO_2_ for hypoxia; 95% O_2_/5% CO_2_ for normoxia) to maintain pH 6.4 and 7.4, respectively. The duration of each phase was optimised per cell type to induce mitochondrial fission during hypoxia and fusion during reoxygenation. Buffer conditions were verified using an ABL90 FLEX PLUS blood gas analyser. To monitor mitochondrial calcium, cells were transfected with mitoGCamP6 and imaged at defined intervals to minimise phototoxicity.

### 2.9. Animal Ethics and Procedures

MiD49 KO mice were initially developed at La Trobe University on a C57BL/6NTac background (http://velocigene.com/komp/detail/10452, accessed on 3 October 2025), and re-established at University College London and Singapore. All procedures at University College London complied with the UK Animals (Scientific Procedures) Act 1986. Singapore studies were approved by the SingHealth Institutional Animal Care and Use Committee (IACUC) under a Responsible Care & Use of Laboratory Animals (RCULA) licence.

### 2.10. Western Blotting

Excised mouse hearts were homogenised and lysed in T-PER buffer (Thermo Fisher Scientific, Waltham, MA, USA) supplemented with protease inhibitors. Protein concentration was determined using a BCA assay, and equal amounts of total protein (20 µg per lane) were resolved by SDS–PAGE alongside a molecular weight ladder. Proteins were transferred onto PVDF membranes using a semi-dry transfer system.

Membranes were blocked in 5% non-fat milk in TBS-T (50 mM Tris-HCl, 150 mM NaCl, 0.1% Tween-20, pH 7.6) for 4 h at room temperature, followed by incubation with primary antibodies against MID49 (Santa Cruz Biotechnology, Dallas, TX, USA; sc-515800; 1:100) and GAPDH (Proteintech, Rosemont, IL, USA; 1:25,000) overnight at 4 °C with gentle agitation. After washing in TBS-T, membranes were incubated with appropriate HRP-conjugated secondary antibodies and developed using chemiluminescent detection.

Protein bands were visualised using a digital imaging system, and band intensities were quantified using ImageJ 2.16.0/1.54p. Uncropped immunoblots, including visible molecular weight markers, are provided in the Supplementary Information [33].

### 2.11. Transmission Electron Microscopy (TEM)

Wild-type and MiD49 KO mice (8–12 weeks) were anaesthetised with isoflurane, followed by cervical dislocation. Hearts were excised, fixed overnight, and processed within 72 h. Ultrathin (90 nm) sections of the left ventricle were imaged using a JEOL JEM 2100 transmission electron microscope at 4000× and 6000× magnifications. Interfibrillar mitochondrial morphology was assessed in randomly selected longitudinal sections of cardiomyocytes and compared to relaxed sarcomeric length (2 µm) [26].

### 2.12. Echocardiography

Male mice aged 8–13 weeks were anaesthetised with 2% vaporised isoflurane in 1 L/min oxygen and placed supine on a heated ECG monitoring platform (Vevo Imaging Station). Anaesthesia was maintained with 0.6–1.2% isoflurane. Transthoracic echocardiography was performed using a Vevo 2100 system with an MS400 18–38 MHz transducer (VisualSonics). M-mode and 2D imaging were acquired in the parasternal short-axis (SAX) and long-axis (PLAX) views. Pulsed-wave Doppler was used to assess aortic arch blood flow velocity. To evaluate cardiac response to stress, low-dose isoproterenol treatment was used to identify irregular cardiac function and contractile reserve, which may not be evident under basal conditions [34]. Imaging was repeated 5 min after intraperitoneal injection of isoproterenol (4 ng/g; 16504, Sigma-Aldrich, St. Louis, MO, USA) [35]. All measurements were performed in triplicate per mouse.

### 2.13. Non-Recovery Acute Ischaemia-Reperfusion In Vivo Model

Anaesthesia for thoracotomy and left anterior descending coronary artery (LAD) ligation was induced with ketamine, 100 mg/Kg, IP, and xylazine, 10 mg/Kg, IP, and maintained with 0.5% isoflurane in air via endotracheal intubation. Pre-operative analgesia consisted of buprenorphine 0.05–0.1 mg/kg s.c., with additional doses given if required. Body temperature was maintained at 37 °C using a heating pad.

A curved 3/8 circle suture needle was inserted into the left ventricle (LV) to pass an 8-0 silk suture under the LAD. Subsequently, a small polyethylene tube was placed on the surface of the LV and tightly tied down with the suture to induce LAD occlusion. Ischaemia resulted in pallor cardiac tissue below the occlusion, reduced LV contraction, and ECG ST-segment elevation. LAD occlusion of WT C57BL/6 mice, for a period of 45 min, has been previously characterised in our laboratory to achieve an infarct size of 45–50%. Reperfusion was achieved by releasing the polyethylene tube, resulting in increased LAD contraction and restoration of the red colour of the myocardium (maintained for a period of 120 min).

For in vivo histological staining to identify the area at risk (AAR), the LAD was re-occluded with the suture running beneath it before perfusion with 0.5% Evans blue. Following staining, the hearts were frozen and sectioned into transverse slices. Hearts slices were incubated in 1% 2,3,5-triphenyltetrazolium chloride (TTC, in PBS) for 30 min at 37 °C to identify infarcted tissue (IS). Fixed sections were imaged and analysed using Image J (version 1.8). Analysis of the percentage of AAR ((LV area- Evans blue-stained area/LV area) ×100) and IS ((IS area/AAR) ×100) was carried out by one observer blinded to the experimental groups.

### 2.14. Statistical Analysis

Data were analysed using GraphPad Prism 10 and are reported as mean ± SEM. Data distribution was assessed for normality, and variance homogeneity was evaluated prior to applying parametric tests. Where assumptions were met, one-way or two-way ANOVA with Bonferroni post hoc tests, or unpaired *t*-tests, were used as appropriate. Statistical significance was defined as *p* < 0.05.

N indicates the number of independent experimental (biological) replicates within each experiment. For individual experimental groups, *n* indicates the number of independent replicates per group. Technical replicates were averaged and not treated as independent observations.

## 3. Results

### 3.1. MiD Knockdown Promotes Mitochondrial Elongation and Protects Cardiac Cells from SIRI

**Figure 1:**
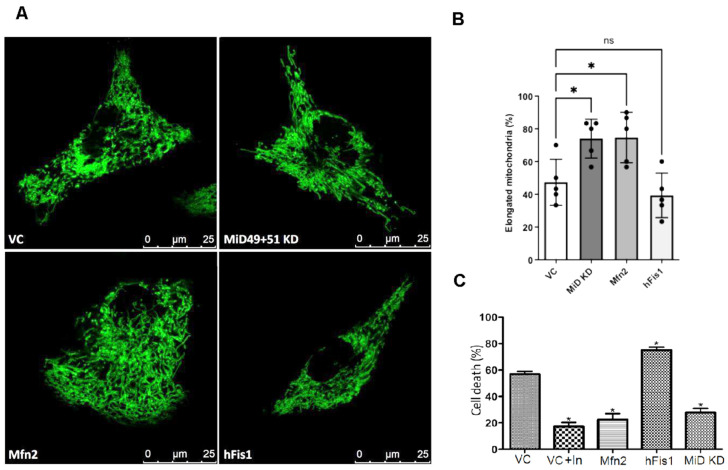
MiD49 and MiD51 knockdown promotes mitochondrial fusion and protects against SIRI-induced cell death in HL-1 cells. (**A**) Representative confocal images of fixed HL-1 cells co-transfected with mtGFP to visualise mitochondrial morphology. Images show: VC cells (top left), MiD49 and MiD51 knockdown (MiD KD, top right), Mfn2 overexpression (bottom left), and hFis1 overexpression (bottom right). Knockdown of MiD49 and MiD51, similar to Mfn2 overexpression, results in a predominantly fused mitochondrial network, in contrast to the fragmented phenotype observed in VC and hFis1-overexpressing cells. (**B**) Quantification of the percentage of cells displaying elongated mitochondria across conditions. MiD KD knockdown and Mfn2 overexpression significantly increased mitochondrial fusion compared to VC, while hFis1 overexpression had no such effect. Statistical analysis: one-way ANOVA (*p* = 0.0012) followed by a Bonferroni post hoc test; N = 5, 150 cells imaged per condition; * *p* < 0.05; ns, not significant. (**C**) Assessment of cell death following simulated ischaemia—reperfusion injury (SIRI) in HL-1 cells. Insulin-treated VC cells, Mfn2-overexpressing cells, and MiD KD cells showed a significant reduction in cell death, while hFis1 overexpression increased cell death compared with untreated VC. Statistical analysis: unpaired *t*-test; N = 6, 300 cells per condition; * *p* < 0.05.

Previous research demonstrated that the knockdown of MiD49 and MiD51 promotes mitochondrial elongation in non-cardiac cell lines [11,12,36,37]. Building on this, we investigated their role in cardiomyocytes by knocking down MiD49 and MiD51 in HL-1 cells using specific shRNA-encoding plasmids. This significantly increased mitochondrial elongation compared to the control (VC) group (Figure 1A), resulting in a predominantly fused morphology similar to that observed with Mfn2 overexpression (74.7 ± 4.6% Mfn2, 74.0 ± 3.6% MiD KD, 47.3 ± 4.2% VC; Figure 1B). In contrast, hFis1 overexpression had no significant effect on mitochondrial morphology (Figure 1A), compared with VC cells (39.3 ± 4.0%; Figure 1B).

Based on these initial observations, we next examined whether mitochondrial elongation could protect against cell death during SIRI. To investigate this, HL-1 cells were subjected to 7 h of simulated ischaemia followed by 1 h of reperfusion using hypoxic and normoxic buffers, respectively, in a hypoxic chamber. Our findings revealed that dual knockdown of MiD49 and MiD51 significantly reduced cell death compared with vector control cells (56.7 ± 2.4% VC vs. 27.9 ± 3.0% MiD KD; Figure 1C), suggesting a protective role for mitochondrial elongation in this context [38].

To corroborate these findings and further confirm the critical involvement of mitochondrial dynamics in SIRI-induced cell death, we employed both pharmacological and genetic strategies to modulate mitochondrial morphology [8,38,39]. We utilised insulin treatment, a well-established cardioprotective intervention known to mimic ischemic postconditioning and activate the reperfusion injury salvage kinase pathway, thereby influencing mitochondrial integrity and reducing cell death [40]. Additionally, we directly manipulated mitochondrial structure by overexpressing Mfn2 to promote mitochondrial fusion [41,42] and overexpressing hFis1 to drive mitochondrial fission [43]. Consistent with the protective effect of mitochondrial elongation, insulin treatment significantly reduced cell death compared with VC cells (17.3 ± 3.0% vs. 56.7 ± 2.4%; Figure 1C). Similarly, Mfn2 overexpression promoted mitochondrial fusion and also lowered cell death (22.3 ± 4.7%). Conversely, overexpression of hFis1 significantly increased cell death (75.2 ± 2.2%), highlighting the detrimental impact of excessive mitochondrial fission during SIRI (Figure 1C).

The observed reduction in cell death within the MiD KO group was comparable to the protection achieved in both insulin-treated cells and those overexpressing Mfn2. This strongly suggests that inhibition of mitochondrial elongation, specifically mediated by MiD49 and MiD51 knockdown, confers significant protection during IRI. These findings collectively highlight the critical role of mitochondrial morphology in determining cell fate during SIRI, with inhibition of mitochondrial fission appearing to consistently promote cell survival [44,45].

### 3.2. MiD49 and MiD51 Knockdown Preserves Mitochondrial Morphology and Calcium Handling During Real-Time SIRI

**Figure 2:**
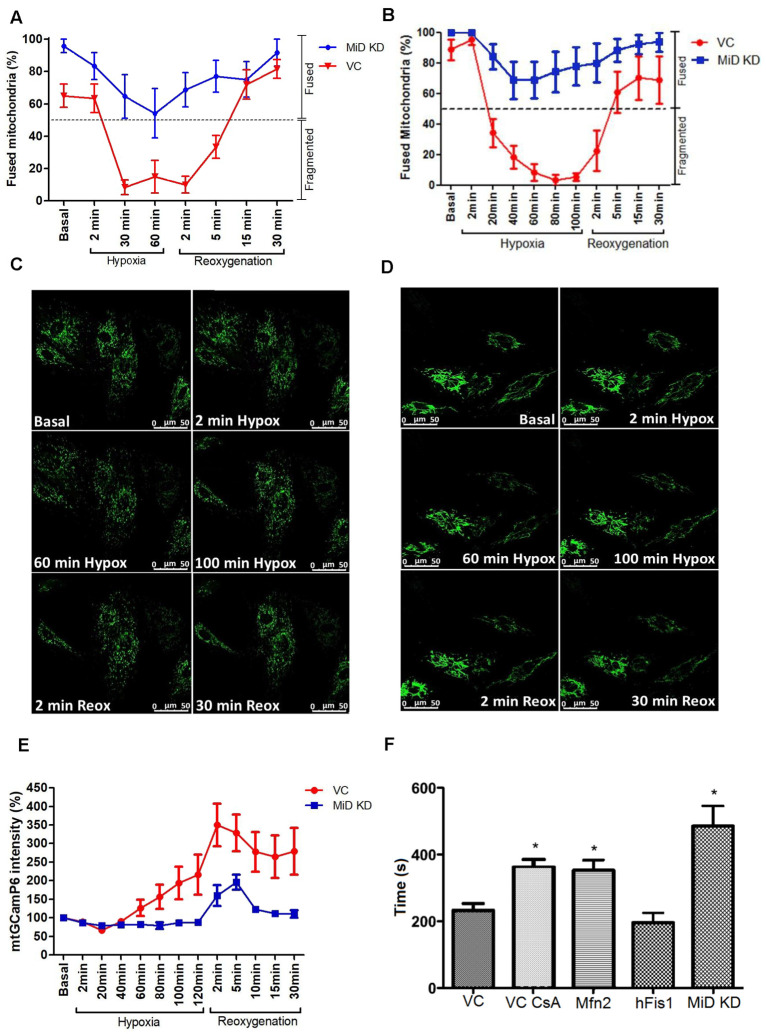
MiD49 and MiD51 knockdown preserve mitochondrial structure, alter calcium handling dynamics and delay MPTP opening. (**A**) Quantification of mitochondrial morphology in HL-1 cells during hypoxia and reoxygenation. Two-way ANOVA revealed a significant Group × Time interaction (*p* = 0.006), indicating distinct temporal mitochondrial responses between vector control (VC) and MiD49/51 knockdown (MiD KD) cells (VC *n* = 10, MiD KD *n* = 8). (**B**) Quantification of mitochondrial morphology in H9c2 cells using the same hypoxia–reoxygenation time course showed a similar pattern. Two-way ANOVA revealed a significant Group × Time interaction (*p* < 0.0001), indicating preservation of mitochondrial fusion in MiD KD cells compared with VC cells across time (N = 6). (**C**,**D**) Representative confocal images of H9c2 cells expressing mtGFP. (**C**) VC cells displayed fragmented mitochondria during hypoxia and partial recovery upon reoxygenation. (**D**) MiD KD cells maintain a predominantly fused mitochondrial network throughout hypoxia and reoxygenation. (**E**) Mitochondrial calcium dynamics during simulated ischaemia-reperfusion injury measured using mtGCaMP6. Two-way ANOVA revealed a significant Group × Time interaction (*p* = 0.0073), indicating distinct temporal mitochondrial Ca^2+^ responses between VC and MiD KD cells (VC *n* = 6, MiD KD *n* = 7). (**F**) Knockdown of MiD49 and MiD51 significantly delayed mitochondrial permeability transition pore (MPTP) opening in HL-1 cells, as measured by laser-induced loss of TMRM fluorescence under oxidative stress. This delay was comparable to that observed in positive control groups, including cells overexpressing Mfn2 or pre-treated with cyclosporin A (CsA). In contrast, overexpression of the pro-fission protein hFis1 did not alter susceptibility to pore opening compared with VC cells (N = 6 per group, 120 cells imaged per condition; one-way ANOVA with Bonferroni post hoc test, * *p* < 0.05 vs. VC).

To further characterise the effects of MiD49 and MiD51 knockdown during SIRI, we utilised a real-time (RT) imaging approach to monitor mitochondrial morphology and calcium dynamics in HL-1 and H9c2 cells under hypoxic and reoxygenation conditions. The duration of hypoxia/reoxygenation was optimised for each cell type (Supplementary Figure S1). In HL-1 cells, VC mitochondria consistently underwent fragmentation during hypoxic conditions, followed by partial recovery during reoxygenation. Notably, hyperfusion was observed during early reoxygenation phases, a typical indicator of mitochondrial stress [10,46] (Figure 2A, Supplementary Figure S1). In contrast, mitochondria morphology in MiD49 and MiD51 knockdown cells maintained a predominantly fused mitochondrial network throughout, with no significant changes in morphology over time (Figure 2A), confirming that MiD knockdown protects against mitochondrial fragmentation during RT SIRI (Supplementary Videos S1A and S1B, respectively).

Similar results were observed in H9c2 cells using the real-time SIRI model. VC cells exhibited significant mitochondrial fragmentation during hypoxia, which began to recover upon reoxygenation. In contrast, MiD49 and MiD51 knockdown prevented this fragmentation, with mitochondria remaining predominantly fused throughout hypoxia and reoxygenation (Figure 2B–D, Supplementary Videos S2A and S2B, respectively). Under simulated normoxic conditions, there was no time-dependent change in mitochondrial morphology in VC or MiD49 and MiD51 knockdown cells, suggesting that the protective effect of MiD knockdown is specific to hypoxic stress (Supplementary Figure S2).

As changes in mitochondrial morphology are known to affect calcium buffering capacity, we examined whether MiD49 and MiD51 knockdown influenced mitochondrial calcium dynamics during hypoxia and reoxygenation. Mitochondrial calcium levels were monitored using the genetically encoded calcium indicator mtGCamP6. Environmental conditions within the real-time chamber were tracked throughout the protocol to confirm consistent SIRI exposure across experimental groups (Supplementary Figure S3). In VC cells, mitochondrial matrix calcium levels increased progressively during hypoxia and peaked during early reoxygenation, consistent with calcium overload and dysregulation. In contrast, there was a significant improvement in calcium handling in the knockdown group. MiD49 and MiD51 knockdown appeared to limit this increase, with calcium levels remaining relatively stable and returning toward baseline during reoxygenation (Figure 2E, Supplementary Table S1). These findings point to a possible protective role of mitochondrial elongation in regulating calcium homeostasis under stress conditions.

### 3.3. MiD49 and MiD51 Knockdown Inhibits MPTP Opening Induced by Oxidative Stress

To further evaluate whether MiD49 and MiD51 knockdown influences mitochondrial function downstream of calcium handling, we assessed mitochondrial permeability transition pore (MPTP) opening under oxidative stress in both HL-1 and H9c2 cardiomyocytes. In HL-1 cells, MiD knockdown and expression of the dominant-negative Drp1 mutant (Drp1^K38A) significantly delayed MPTP opening compared with vector control (VC), while individual MiD49 or MiD51 knockdown produced intermediate, non-significant effects. Overexpression of the fission factor Mff accelerated MPTP opening, consistent with increased susceptibility to oxidative injury (Supplementary Figure S4A). In H9c2 cells, pharmacological inhibition of MPTP with cyclosporin A (CsA) confirmed assay sensitivity by significantly delaying pore opening relative to VC, whereas MiD knockdown and Mfn2 overexpression showed only a trend toward delayed opening that did not reach statistical significance (Supplementary Figure S4B).

Collectively, these results indicate that suppression of mitochondrial fission, either via MiD49/51 silencing or Drp1 inhibition, can delay MPTP opening, thereby linking mitochondrial elongation and calcium stabilisation to improved resistance against oxidative stress.

### 3.4. MiD49 Deletion Increases the Proportion of Elongated Mitochondria in Cardiomyocytes In Vivo

Our in vitro data demonstrated that simultaneous knockdown of MiD49 and MiD51 promoted mitochondrial elongation, stabilised calcium dynamics, and delayed MPTP opening during SIRI, highlighting the functional role of MiDs proteins in maintaining mitochondrial integrity under stress conditions. To translate these findings into an in vivo context, we focused on MiD49 due to its distinct roles in mitochondrial regulation [10,47,48]. Knockdown of MiD49 has been shown to reduced doxorubicin-induced mitochondrial fission and apoptosis in cardiomyocytes, as well as to protect the heart from doxorubicin-induced cardiotoxicity [49]. Additionally, MiD49 does not dimerise [47], a unique structural feature that provides greater flexibility in responding to cellular stress. In contrast, double knockout of MiD49 and MiD51 is embryonically lethal [48], further supporting the rationale for focusing on MiD49 alone.

**Figure 3:**
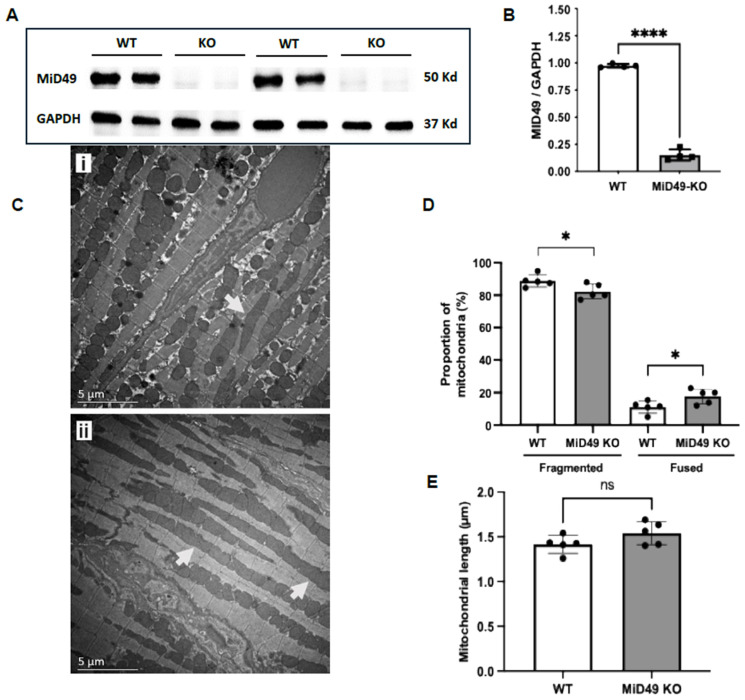
Validation of MiD49 knockout and assessment of mitochondrial morphology in the hearts of MiD49 KO mice. (**A**) Representative Western blot showing MiD49 protein expression in heart tissue from WT and MiD49 KO mice, with GAPDH as the loading control. (**B**) Quantification of MiD49 protein levels normalised to GAPDH. MiD49 expression was significantly reduced in KO hearts compared with WT (WT: 0.979 ± 0.009; KO: 0.128 ± 0.008; *p* = 0.0001; N = 4; unpaired *t*-test). (**C**) Representative transmission electron microscopy (TEM) images of interfibrillar mitochondria in WT (i) and MiD49 KO (ii) left ventricular cardiomyocytes under basal conditions. Representative elongated mitochondria indicated by arrows. (**D**) Mitochondrial morphology was assessed by comparing mitochondrial length to sarcomere length during relaxation (2 µm). MiD49 KO hearts exhibited a significantly higher proportion of fused mitochondria compared to WT under basal conditions, (*p* = 0.0384; N = 5), indicating a shift toward mitochondrial elongation. (**E**) Quantification of mean mitochondrial length showed no statistically significant difference between WT and MiD49 KO cardiomyocytes (*p* = 0.1251; N = 5; unpaired *t*-test). Asterisks represent significance levels (* *p* < 0.05; **** *p* < 0.0001; ns, not significant).

To investigate the role of MiD49 in mitochondrial dynamics in vivo, we utilised a global MiD49 knockout (KO) transgenic mouse model (Figure 3A,B). Baseline mitochondrial morphology was assessed in left ventricular (LV) cardiomyocytes using transmission electron microscopy (TEM). MiD49 KO cardiomyocytes exhibited a significantly higher proportion of elongated mitochondria compared with wild-type (WT) controls (WT: 11.1 ± 1.7% vs. MiD49 KO: 17.7 ± 2.0%; *p* < 0.05; Figure 3C,D). However, there was no statistically significant difference in mean mitochondrial length between the two groups (WT: 1.4 ± 0.05 µm vs. MiD49 KO: 1.5 ± 0.06 µm; *p* = 0.1251; Figure 3E).

### 3.5. Deletion of MiD49 Does Not Affect Cardiac Response to Ischaemia-Reperfusion Injury

**Figure 4:**
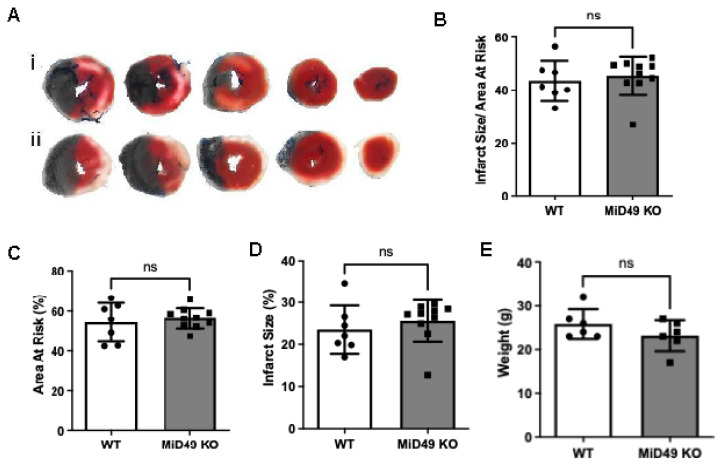
Deletion of MiD49 does not confer protection against myocardial ischaemia–reperfusion injury. (**A**) Representative triphenyltetrazolium chloride (TTC)-stained left ventricular cross-sections from wild-type (WT; i) and MiD49 knockout (KO; ii) mice subjected to 45 min of left anterior descending (LAD) coronary artery occlusion followed by 2 h of reperfusion. White areas indicate infarcted tissue, red represents viable myocardium within the area at risk (AAR), and blue demarcates non-ischaemic regions. (**B**) Quantification of infarct size as a percentage of the area at risk (IS/AAR %). (**C**) Quantification of the area at risk (AAR %) relative to the left ventricle (LV). (**D**) Infarct size expressed as a percentage of total LV area (IS %). (**E**) Body weight comparison between WT and MiD49 KO mice at the time of surgery. Data are presented as mean ± SEM; *n* = 7–10 per group. Statistical significance was assessed by unpaired *t*-test or Mann–Whitney test as appropriate; ns, not significant.

To assess whether MiD49 deficiency alters cardiac susceptibility to myocardial infarction, 11–12-week-old male MiD49 knockout mice and their wild-type littermates were subjected to non-recovery ischaemia-reperfusion (IRI) surgery, in which the left anterior descending (LAD) coronary artery was occluded for 45 min, followed by 2 h of reperfusion. Quantitative analysis revealed no significant differences between MiD49 KO and WT mice in infarct size (IS, % of left ventricle; WT: median 22.0%, *n* = 7; KO: median 27.2%, *n* = 10; *p* = 0.1613, Mann–Whitney test). The area at risk (AAR, % of LV) was also comparable between groups (WT: 54.23%, *n* = 7; KO: 56.23%, *n* = 10; *p* = 0.5883, unpaired *t*-test). Similarly, the infarct-to-risk ratio (IS/AAR, %) did not differ significantly between groups (WT: median 41.28%, *n* = 7; KO: median 47.57%, *n* = 10; *p* = 0.3148, Mann–Whitney test). These findings were accompanied by no significant differences in body weight (WT: 25.83 g, *n* = 6; KO: 23.17 g, *n* = 6; *p* = 0.2149, unpaired *t*-test), suggesting that both groups were physiologically similar at baseline (Figure 4A–E).

### 3.6. MiD49 Knockout Mice Exhibited Similar Cardiac Function to Wild-Type Under Basal and Adrenergic Stress Conditions

To assess whether MiD49 deficiency influences cardiac performance under a physiological stress challenge, we evaluated left ventricular (LV) structure and function following β-adrenergic stimulation.

**Figure 5:**
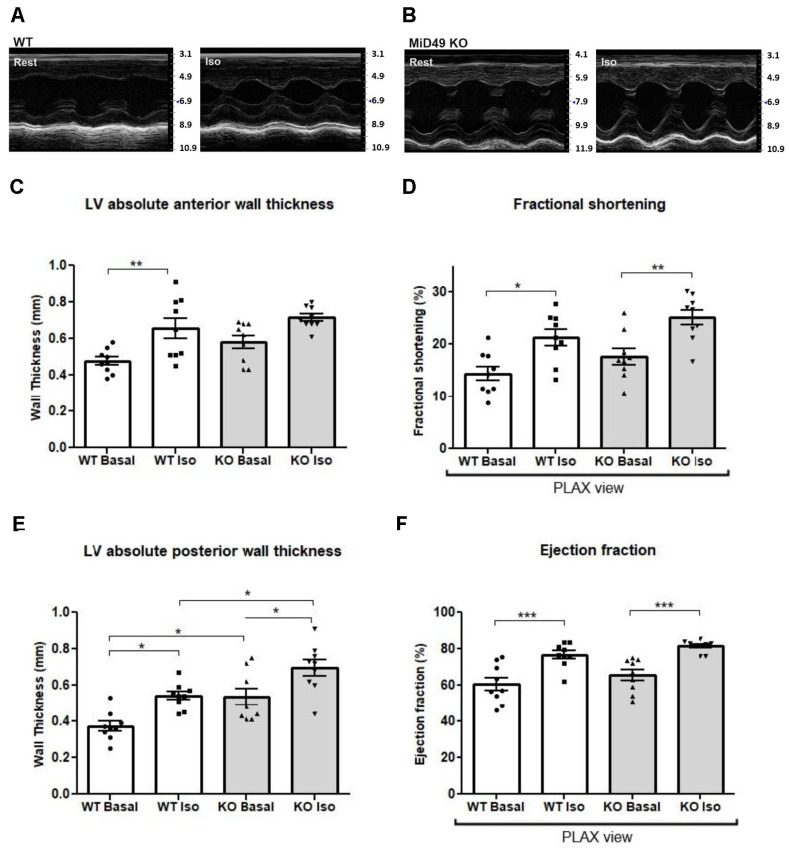
MiD49 knockout mice exhibit comparable cardiac function to wild-type under basal and β-adrenergic stress conditions. (**A**,**B**) Representative M-mode echocardiographic images in the parasternal long-axis (PLAX) view from (**A**) wild-type (WT) and (**B**) MiD49 knockout (KO) mice at rest and following intraperitoneal isoproterenol (Iso, 4 ng/g) administration. (**C**) Left ventricular (LV) absolute anterior wall thickness at basal and under β-adrenergic stimulation. (**D**) Fractional shortening (FS %) and (**F**) ejection fraction (EF %) in WT and MiD49 KO mice before and after Iso challenge. (**E**) LV absolute posterior wall thickness. Both genotypes showed the expected increase in wall thickness and systolic function after isoproterenol. Significant differences between WT and MiD49 KO groups only observed in LV posterior wall thickness. Data are expressed as mean ± SEM; *n* = 6–10 mice per group. * *p* < 0.05; ** *p* < 0.01; *** *p* < 0.001.

Cardiac morphology and function were examined in 8-13-week-old MiD49 KO and wild-type mice (Figure 5A,B) using M-mode echocardiography in the parasternal short-axis view. Enlarged echocardiographic images are provided in Supplementary Figure S9). As expected, isoproterenol treatment induced a significant increase in LV anterior and posterior wall thickness in both genotypes compared with baseline (Figure 5C,E; Supplementary Figure S5B,D). At baseline, MiD49 KO mice exhibited a modest but significant increase in posterior wall thickness relative to WT under basal conditions and following isoproterenol administration (Figure 5E, Supplementary Figure S5D). LV mass did not differ significantly between genotypes either at baseline or post-treatment (Supplementary Figure S5E).

Functional parameters, including heart rate, stroke volume, cardiac output, and peak aortic flow velocity, were comparable between MiD49 KO and WT mice at baseline and after isoproterenol exposure (Supplementary Figure S6A–D). Although minor intra-group fluctuations were observed, isoproterenol stimulation did not produce statistically significant within-group changes in these parameters.

Importantly, there were no significant differences in left ventricular ejection fraction (LVEF) or fractional shortening (LVFS) between WT and MiD49 KO mice at baseline (Figure 5D,F, Supplementary Figure S7A–D). Isoproterenol treatment significantly increased both LVEF and LVFS in both groups, indicating preserved cardiac contractile reserve and normal adrenergic responsiveness independent of MiD49 status.

## 4. Discussion

This study demonstrates that while simultaneous knockdown of MiD49 and MiD51 in vitro promoted mitochondrial elongation, stabilised calcium handling, delayed MPTP opening, and enhanced cardiomyocyte survival during SIRI, deletion of MiD49 alone in vivo did not alter cardiac function (during basal or β-adrenergic stress stimulation) or affect myocardial infarct size. These findings indicate that MiD49-dependent mitochondrial elongation alone is insufficient to confer cardioprotection in vivo, despite substantial evidence that promoting mitochondrial fusion or inhibiting fission can attenuate IRI in other settings, for example, through Mfn2 overexpression or pharmacological inhibition of Drp1–receptor interactions with compounds such as M1 [18]. These findings highlight a disconnect between mitochondrial elongation and cardioprotection in vivo. This emphasises the complexity of translating in vitro mechanisms to whole organisms and suggests that the context, timing and mode of manipulating mitochondrial dynamics are critical determinants of outcome. One potential explanation lies in systemic compensatory mechanisms, such as upregulation of MiD51 or other fission-related proteins, which may preserve mitochondrial fission dynamics and blunt any benefit from MiD49 deficiency. Recent evidence identifies MiD51 as the dominant Drp1 receptor during cardiac stress, whose upregulation promotes Drp1 recruitment to mitochondria and exacerbates ischaemia-reperfusion (I/R) injury [23].

Further supporting this idea, metabolic activation of the fission machinery has been shown to involve long-chain acyl-coenzyme A (LC-CoA)-induced oligomerisation of MiD49 and MiD51, enhancing Drp1-mediated fission under nutrient or oxidative stress [50]. These findings underscore the functional redundancy and adaptability of mitochondrial dynamics proteins and suggest that targeting MiD51 may yield more robust therapeutic outcomes in I/R and related conditions.

While fused mitochondrial morphology is often associated with improved calcium buffering, reduced oxidative stress, and decreased apoptosis during cellular stress [8,44,51,52], effective protection at the whole-organ level likely requires preservation of mitochondrial respiration, membrane potential, and bioenergetic efficiency—factors not directly assessed in this study. In line with this, excessive suppression of fission can impair mitophagy and mitochondrial quality control in cardiomyocytes, potentially offsetting the benefits of hyperfusion [53,54,55].

Beyond expression changes, post-translational modifications of Drp1, including phosphorylation, SUMOylation, ubiquitination, and S-nitrosylation, have been shown to critically influence its activity, localisation, and role in cardiac stress responses [10,56,57]. Such regulation may underlie the differential outcomes observed between acute pharmacological inhibition and chronic genetic deletion of fission components.

Limitations of this study include the use of a whole-body MiD49 KO model, which may mask cardiac-specific effects due to developmental or systemic compensatory mechanisms. The embryonic lethality of MiD49/51 double-KO mice also restricted our ability to examine their combined roles in vivo. Although cardiac function was preserved in MiD49-deficient mice during basal and stress conditions, suggesting no major metabolic impairment at the organ level, direct assessment of mitochondrial function would have been informative to rule out subtle effects. In addition, despite the lack of effect of MiD KO on cardiac function and infarct size, we cannot exclude effects of MiD49 ablation on haemodynamic parameters and survival, as these were not measured. It should be noted that isoproterenol was administered as a bolus rather than as a continuous infusion; therefore, we cannot exclude the possibility that peak inotropic effects were missed. This represents a methodological limitation and may have contributed to the lack of detectable changes in stroke volume despite observed increases in absolute wall thickness. Our cell studies did not include assessments of mitochondrial function such as respiration, reactive oxygen species (ROS) production, or ATP synthesis, leaving the functional consequences of mitochondrial elongation to be studied. Consequently, variations in mitochondrial respiratory or metabolic capacity may have resulted in differences in the effective ischaemic or hypoxic burden between experimental conditions. While the present study examined infarct size at 2 h following reperfusion, longer-term analyses of survival and post-infarction remodelling would strengthen the translational relevance of the findings and should be addressed in future studies. In addition, the 2 h reperfusion time point may not provide an accurate representation of infarct size, and assessment at 24 or 72 h may have been more suitable. Furthermore, we did not profile other key mitochondrial dynamics proteins (Drp1, phospho-Drp1, Mfn1, Mfn2, OPA1, MiD51, Mff and Fis1) by Western blot in MiD49 KO versus WT hearts, which would help determine whether compensatory remodelling of the fission and fusion machinery contributes to the absence of cardioprotection in vivo.

To bridge these gaps, future studies should employ tissue-specific or inducible gene-editing strategies (e.g., CRISPR–Cas9 or AAV-mediated systems) to dissect the cardiac-specific roles of MiD49 and MiD51 in a temporally controlled manner. Investigating these proteins in aged or metabolically stressed models may also uncover subtle protective phenotypes not evident in young, healthy animals. Complementary approaches, including high-resolution respirometry, real-time calcium imaging, and three-dimensional electron microscopy, could provide a more holistic view of how mitochondrial dynamics influence cardiac resilience under stress.

Clinically, these insights highlight both the promise and the complexity of targeting mitochondrial dynamics as a therapeutic strategy. Modulating MiD51–Drp1 interactions, potentially through reversible tissue-specific pharmacological inhibitors, may help preserve mitochondrial integrity and improve outcomes following myocardial infarction and other ischaemic events [23]. Emerging pharmacological inhibitors that disrupt MiD51 interactions with other mitochondrial dynamics proteins are under investigation [58]. Early Drp1 inhibitor formulations have already reduced infarct size in murine but not in pig I/R models, supporting the importance of this pathway [59,60]. However, such interventions must carefully balance promoting mitochondrial fusion with maintaining adequate energy production and cellular homeostasis [55].

## 5. Conclusions

Our study suggests that coordinated inhibition of MiD49 and MiD51 may be required to confer cardioprotection rather than targeting the individual MiD proteins alone. These findings underscore the importance of integrating structural, functional, and systemic perspectives when evaluating mitochondrial-targeted therapies and provide a foundation for refining future strategies aimed at modulating mitochondrial dynamics in ischaemic heart disease.

## Data Availability

The original contributions presented in this study are included in the article/Supplementary Material. Further inquiries can be directed to the corresponding author.

## Supplementary Materials

The following supporting information can be downloaded at: https://www.mdpi.com/article/10.3390/cells15060559/s1, Table S1: Time point comparisons of mitochondrial matrix calcium dynamics in VC and MiD KD cells. Figure S1: Characterisation of RT SIRI was carried out to identify the period of hypoxia required to induce mitochondrial fragmentation in VC cells, expressing mtGFP, followed by reoxygenation. Figure S2: Comparison of mitochondrial morphology in VC and MiD49/51 knockdown H9c2 cells under normoxic conditions. Figure S3: Comparable oxygen, glucose, and lactate profiles in VC and MiD49/51 KD cells during RT-SIRI. Figure S4: Mitochondrial permeability transition pore opening in H9c2 and HL-1 cardiomyocytes following modulation of mitochondrial fission. Figure S5: Left ventricular anatomical parameters of WT and MiD49 KO mice assessed by echocardiography. Figure S6: Haemodynamic parameters of WT and MiD49 KO mice under basal and β-adrenergic stimulation. Figure S7: Left ventricular fractional shortening and ejection fraction of WT and MiD49 KO mice measured in short- and long-axis views. Figure S8: Uncropped immunoblots showing MID49 protein expression in wild-type and MID49 knockout mice. Figure S9: Enlarged echocardiographic images of wild-type and MiD49 knockout mice. Video S1A,B: HL-1 VC simulated IRI and HL-1 MiD KD simulated IRI. Video S2A,B: H9c2 VC RT simulated IRI and H9c2 MiD KD RT simulated IRI.

## Abbreviations

2D imaging	Two-dimensional echocardiography imaging
AAR	Area at risk
AAV9	Adeno-associated virus serotype-9
AMI	Acute myocardial infarction
CO	Cardiac output
CVD	Coronary artery disease
Drp1	Dynamin-related protein 1
EDD	End-diastolic diameter
EDV	End-diastolic volume
ER	Endoplasmic reticulum
ESD	End-systolic diameter
ESV	End-systolic volume
FS	Fractional shortening
HR	Heart rate
IRI	Ischaemia-reperfusion injury
IS	Infarct size
KD	Knockdown
KO	Knockout
LAD	Left descending coronary artery
LV	Left ventricle
MI	Myocardial infarction
MiD49	Mitochondrial dynamics proteins of 49 kDa
MiD51	Mitochondrial dynamics proteins of 51 kDa
MiDs	Mitochondrial dynamic proteins MiD49 and MiD51
M-mode	Motion mode
MPTP	Mitochondrial permeability transition pore
mtBFP	Mitochondrial blue fluorescent protein
mtDNA	Mitochondrial DNA
mtGFP	Mitochondrial green fluorescent protein
mtRFP	Mitochondrial red fluorescent protein
OMM	Outer mitochondrial membrane
PLAX	Parasternal long-axis mid-level
RI	Reperfusion injury
ROS	Reactive oxygen species
SAX	Parasternal short-axis view
SI	Simulated ischaemia
SR	Sarcoplasmic reticulum
SV	Stroke volume
TTC	Triphenyl tetrazolium chloride solution
VC	Empty vector control
WT	Wild-type
