# Supplementary material for: Mitochondrial Dynamic Proteins MiD49 and MiD51 as Novel Targets of Cardioprotection
Source: Cells. 2026 Mar 20;15(6):559. doi: 10.3390/cells15060559 (PMC13024988; doi:10.3390/cells15060559)
Supplement: Supplementary file 1 [file cells-15-00559-s001.zip › cells-4046392 Supplementary Figures with figure legends.pdf]

Supplementary Figures

Supplementary Figure S1.

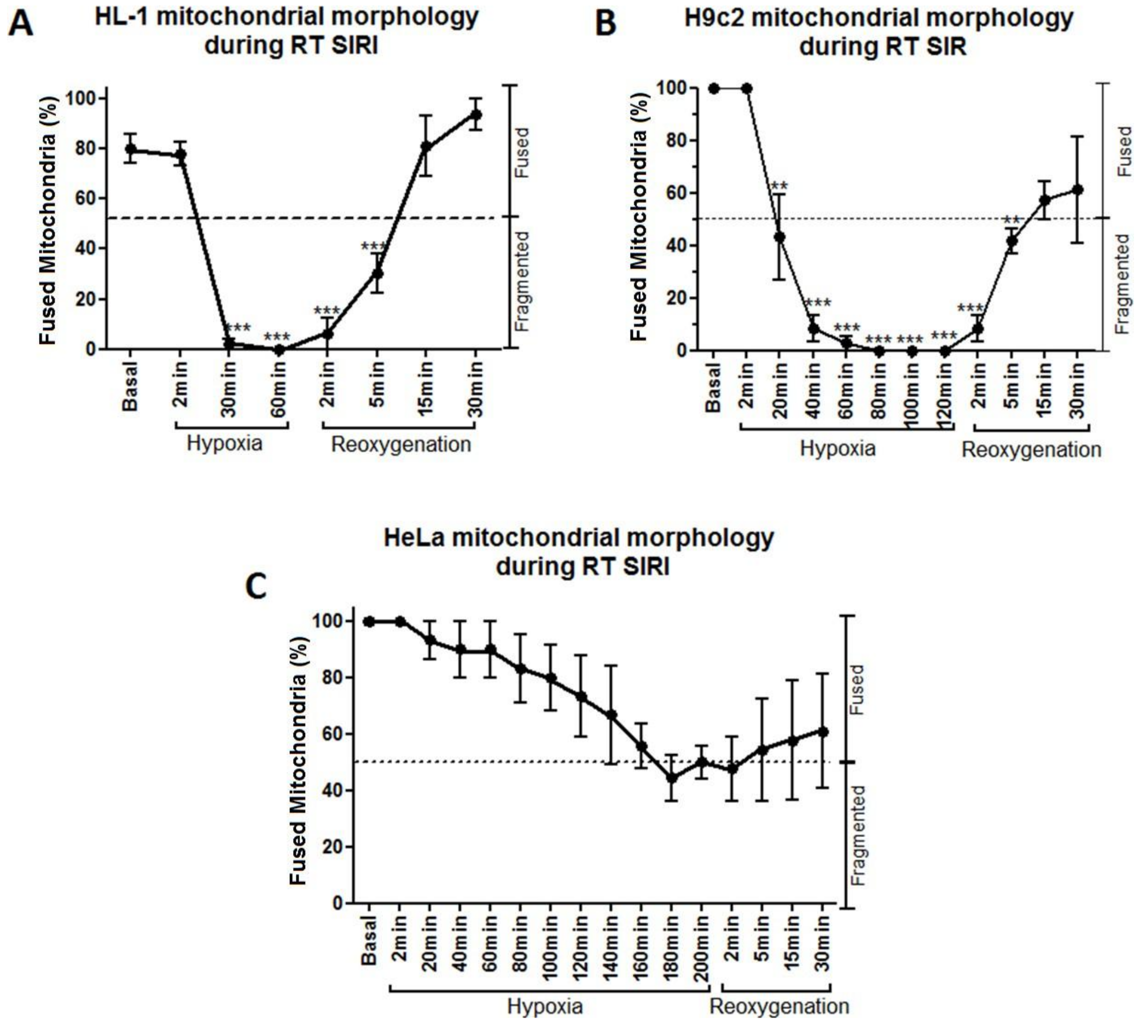

Figure S1

Characterisation of RT SIRI was carried out to identify the period of hypoxia required to induce mitochondrial fragmentation in VC cells, expressing mtGFP, followed by reoxygenation. Periodic imaging was carried out during continuous flow of hypoxic buffer (simulated ischaemia), followed by a period of reoxygenation using normoxic buffer (simulated reperfusion). A) HL-1 cells underwent full mitochondrial fragmentation after 60 minutes of hypoxia. (N=3, P<0.0001). B) 80 minutes of hypoxia was required to induce full mitochondrial fragmentation in H9c2 cells (N=3, P<0.0001). C) There was a gradual increase in mitochondrial fragmentation, but full mitochondrial fragmentation during hypoxia could not be achieved in all HeLa cells, using this model for the same hypoxic times. Cardiac cell models, which rely heavily on oxidative metabolism, demonstrate significantly greater sensitivity to hypoxic injury and loss of viability compared to tumor-derived or cancer-like cell lines (Kuznetsov et al., 2015).

Supplementary Figure S2.

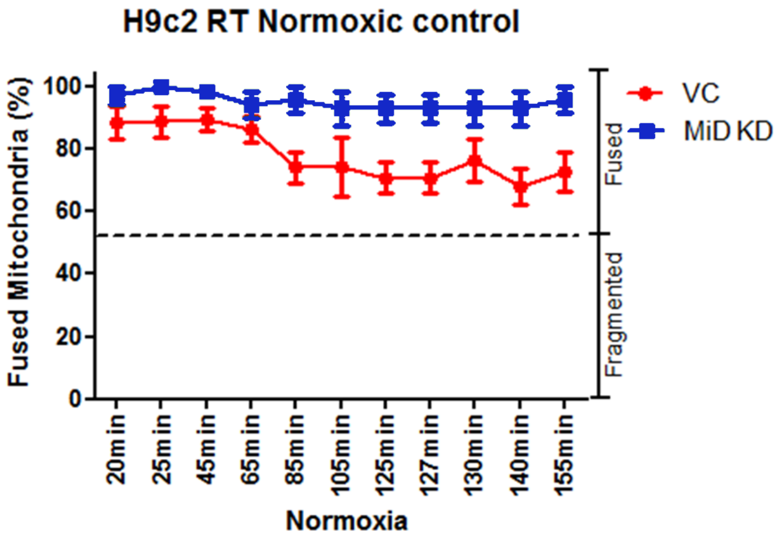

Figure S2. Comparison of Mitochondrial Morphology in VC and MiD49/51 Knockdown H9c2 Cells Under Normoxic Conditions.

In H9c2 cells there was no time-dependent change in mitochondrial morphology between VC and MiD49/51 knockdown cells during normoxia (N = 6; two-way ANOVA).

Supplementary Figure S3.

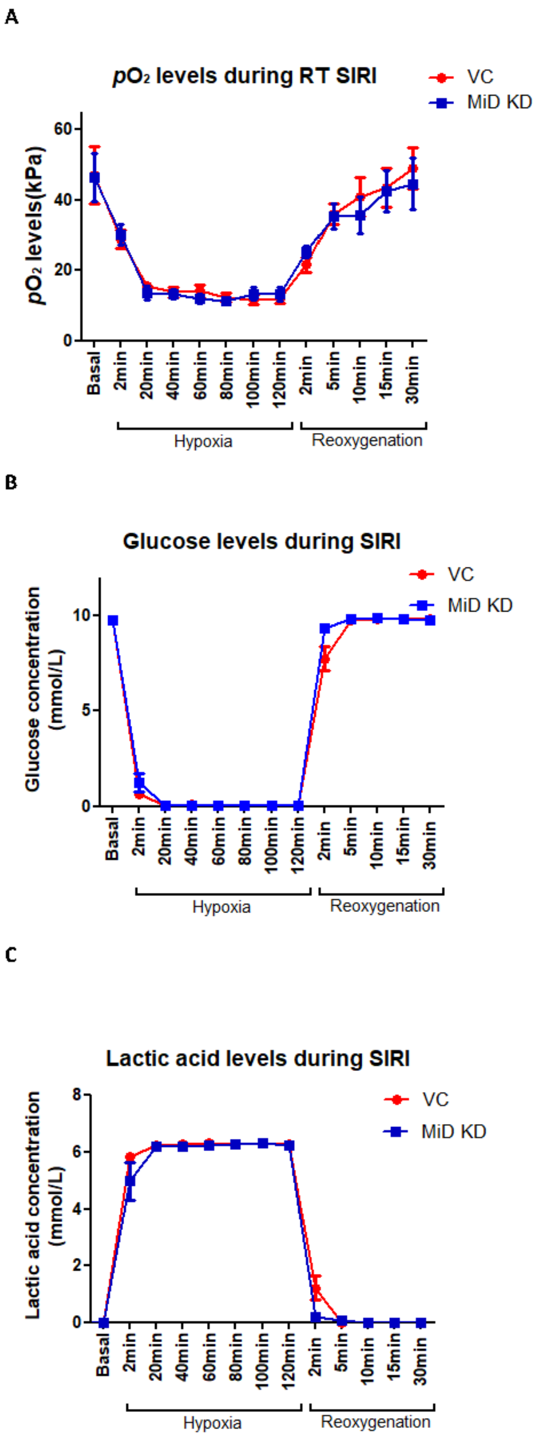

Figure S3. Comparable Oxygen, Glucose, and Lactate Profiles in VC and MiD49/51 KD Cells During RT-SIRI.

Buffer from the RT confocal chamber was collected at each time point during the H9c2 RT-SIRI protocol (Figure 3F) to confirm achievement of hypoxia and reoxygenation (VC *n*= 7, MiD49/51 KD *n*= 6). Oxygen (A), glucose (B), and lactate (C) levels were comparable between groups across time (two-way ANOVA). Measurements were performed using a blood gas analyser (ABL90 FLEX PLUS Blood Gas Analyser, Radiometer).

Supplementary Figure S4.

A

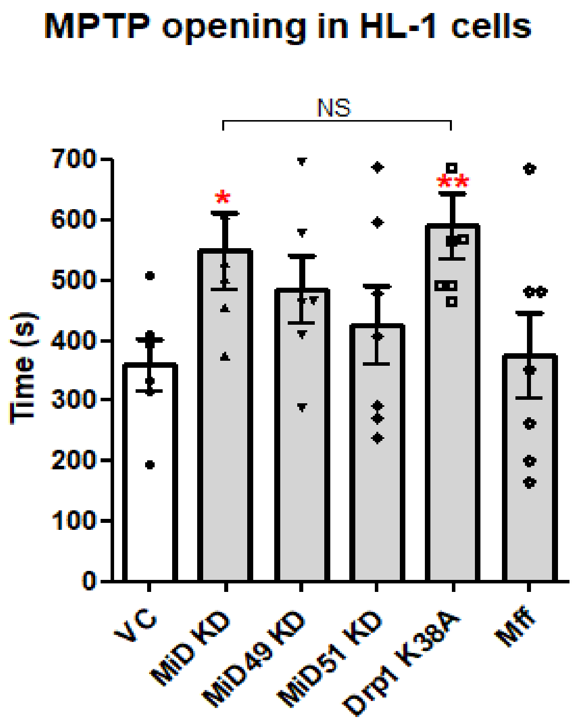

B

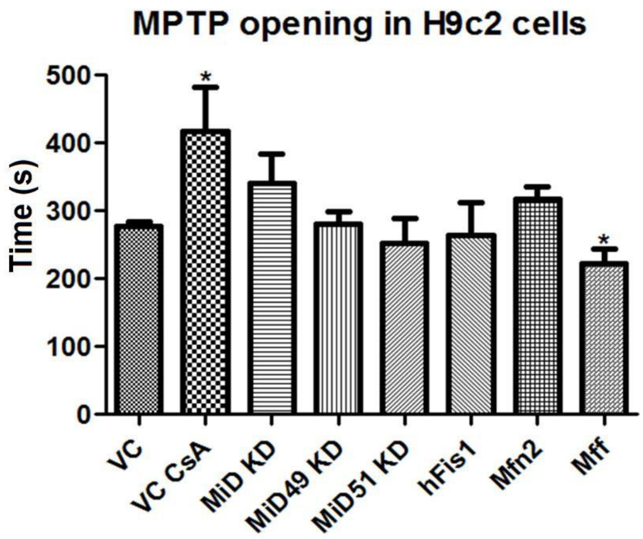

Figure S4. Mitochondrial permeability transition pore opening in H9c2 and HL-1 cardiomyocytes following modulation of mitochondrial fission.

(A) In HL-1 cells, both MiD49/51 knockdown (MiD KD) and expression of the dominant-negative Drp1 mutant (Drp1^K38A) significantly delayed MPTP opening relative to VC, while individual MiD49 or MiD51 knockdown produced intermediate, non-significant effects. Mff overexpression shortened the time to pore opening. (B) In H9c2 cells, pharmacological inhibition of MPTP with cyclosporin A (CsA) significantly delayed pore opening compared with vector control (VC), confirming assay sensitivity. Overexpression of the fission factor Mff accelerated pore opening, whereas MiD49/51 knockdown and Mfn2 overexpression showed a non-significant trend toward delayed opening. Data are expressed as mean ± SEM (N = 5–6 per group). Statistical analysis was performed using one-way ANOVA followed by Bonferroni post-hoc test; *P < 0.05, *_P < 0.01 vs VC; NS, not significant_

Supplementary Figure S5.

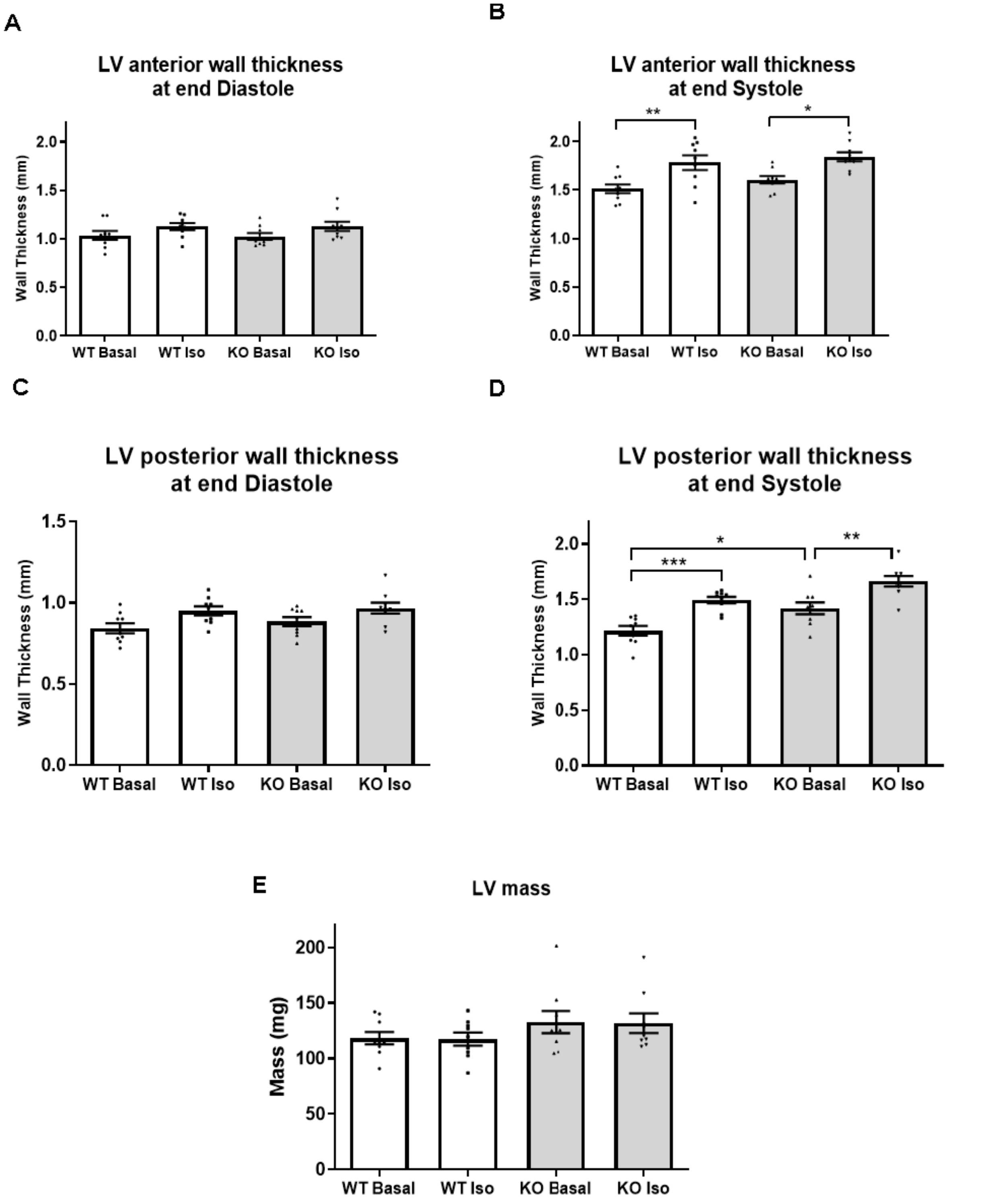

Figure S5. Left ventricular anatomical parameters of WT and MiD49 KO mice assessed by echocardiography.

WT and MiD49 KO anatomical measurements of the left ventricle (LV) were obtained using M-mode echocardiography (N = 9 per group). LV anterior and posterior wall thicknesses at end-diastole did not differ between genotypes at baseline or after isoproterenol stimulation (A, C). At end-systole, LV posterior wall thickness was significantly higher in MiD49 KO hearts at baseline compared with WT (D, $P < 0.05$), but this difference was abolished after isoproterenol treatment (4 ng/g), which increased wall thickness in both groups (B, D, $P < 0.05$). LV mass was comparable between genotypes under all conditions (E). Statistical significance was determined by one-way ANOVA followed by Bonferroni post-hoc test.

Supplementary Figure S6.

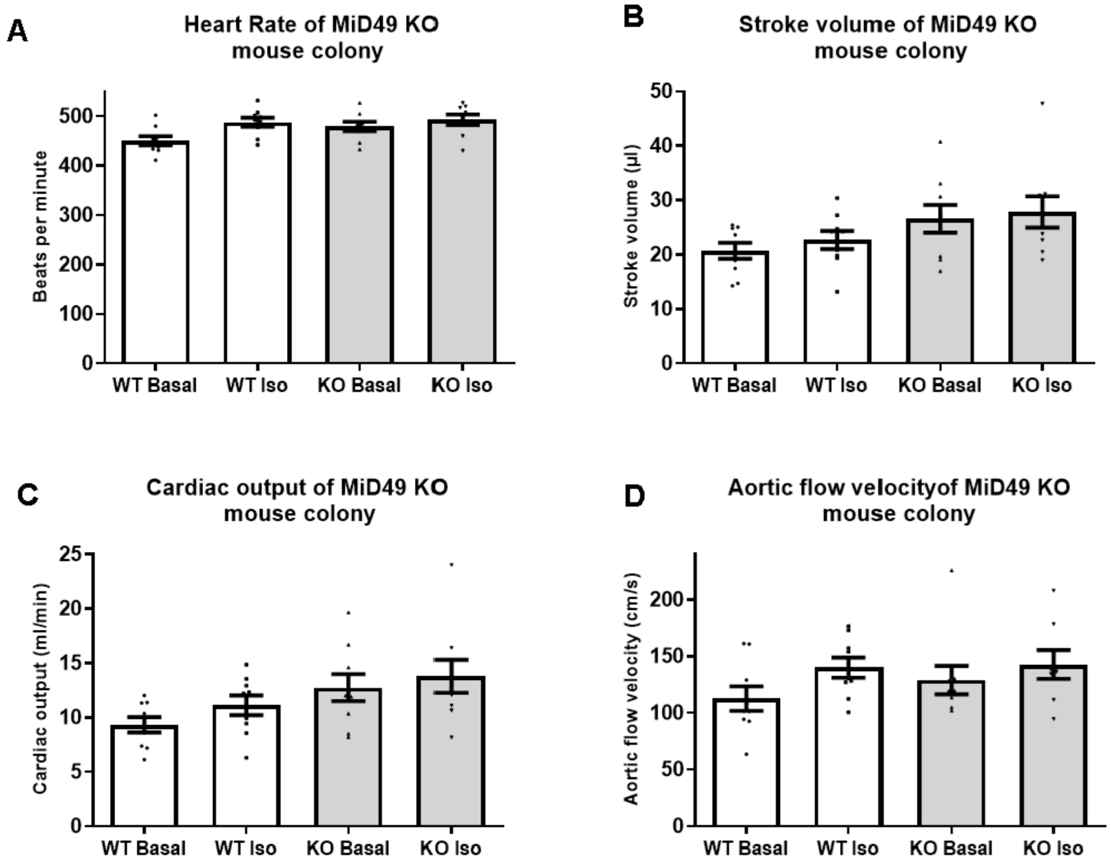

Figure S6. Hemodynamic parameters of WT and MiD49 KO mice under basal and β-adrenergic stimulation.

Heart rate (A), stroke volume (B), cardiac output (C), and peak aortic flow velocity (D) were measured in WT and MiD49 KO mice at baseline and following 4 ng/g isoproterenol stimulation (N = 9 per group). No significant differences were detected between genotypes or treatment conditions ($P > 0.05$). Statistical analysis was performed by one-way ANOVA with Bonferroni post-hoc test comparing WT basal with KO basal, WT basal with WT Iso, and KO basal with KO Iso.

Supplementary Figure S7.

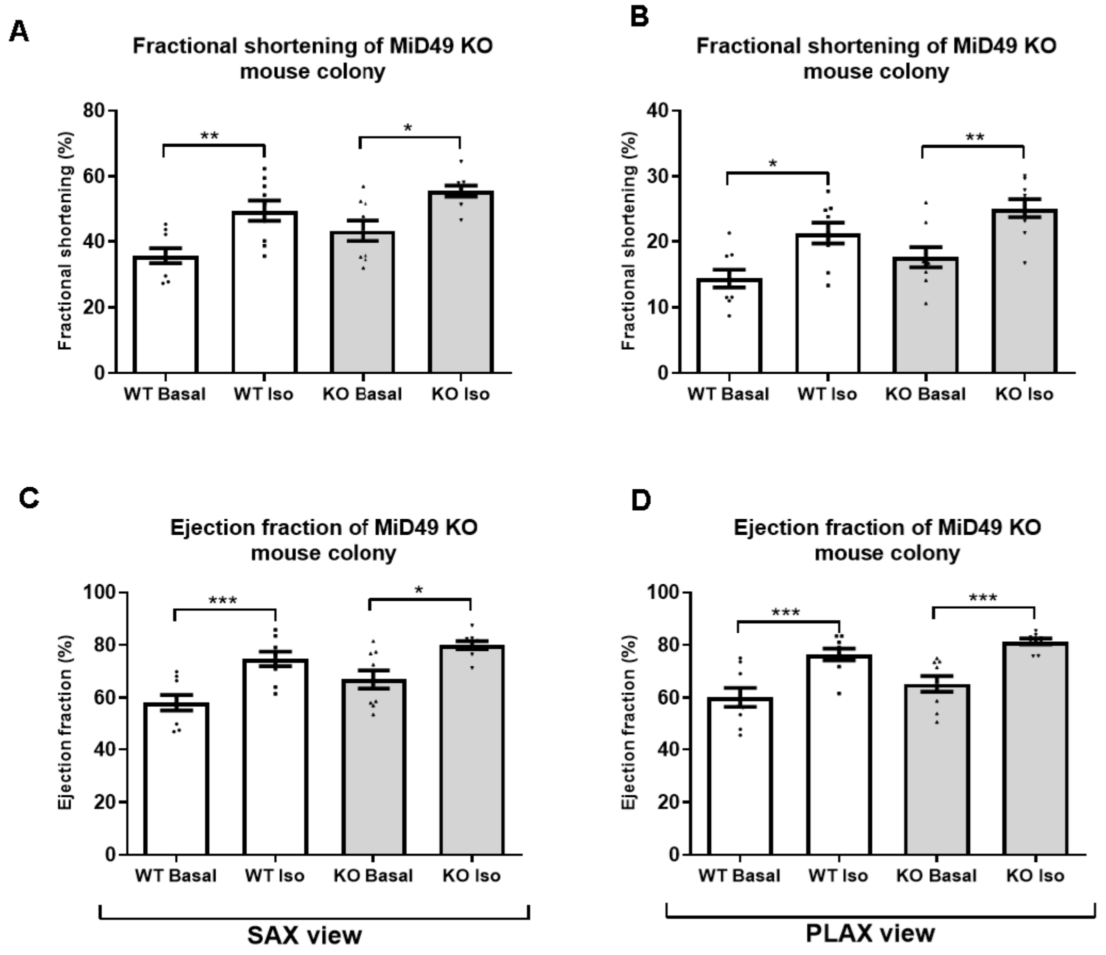

Figure S7. Left ventricular fractional shortening and ejection fraction of WT and MiD49 KO mice measured in short- and long-axis views.

Left ventricular fractional shortening (LVFS) and ejection fraction (LVEF) were determined in WT and MiD49 KO mice using M-mode echocardiography in short-axis (SAX, A and C) and long-axis (PLAX, B and D) views (N = 9 per group). At baseline, LVFS did not differ significantly between genotypes (WT 35.71 ± 2.3 %; KO 43.26 ± 3.1 %). Isoproterenol treatment significantly increased LVFS in both genotypes (WT Iso 49.54 ± 3.3 %; KO Iso 55.61 ± 1.7 %). LVEF values from both SAX and PLAX views followed the same pattern, with no significant genotype differences but a significant increase after isoproterenol stimulation ($P < 0.05$). Statistical analysis was performed by one-way ANOVA with Bonferroni comparison test between WT basal vs KO basal, WT basal vs WT Iso, and KO basal vs KO Iso.

Supplementary Figure S8.

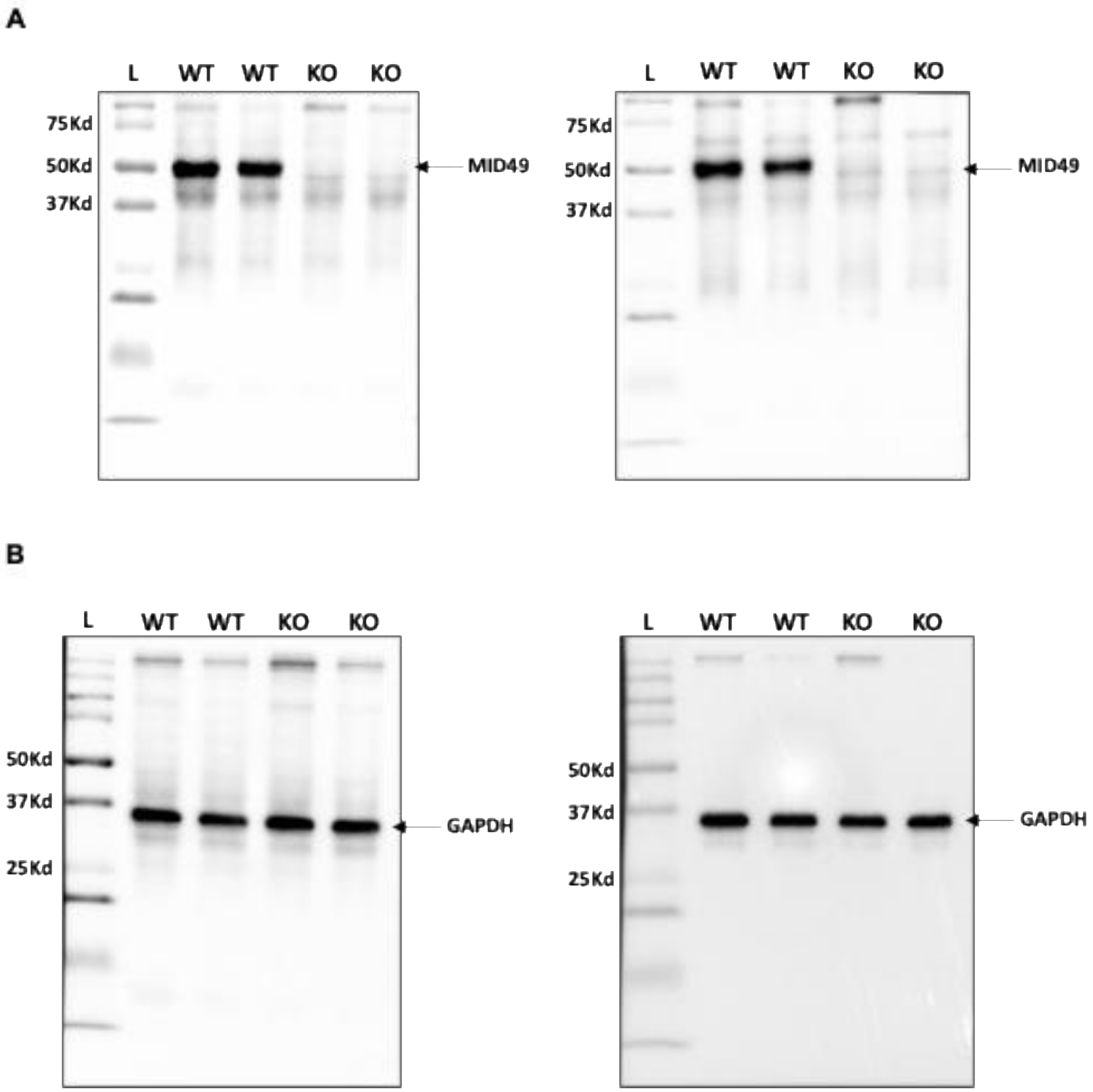

Figure S8. Uncropped immunoblots showing MID49 protein expression in wild-type and MID49 knockout mice. (A) Representative uncropped immunoblots probed for MID49 in protein lysates from wild-type (WT) and MID49 knockout (KO) mouse tissue. Two independent blots are shown. MID49 signal is detected at the expected molecular weight in WT samples and is absent in KO samples. (B) Uncropped immunoblots probed for GAPDH, used as a loading control, for the same samples shown in (A). Representative immunoblots from two independent experiments, comprising a total of n = 4 mice per group, are shown. For all blots, 20 µg of total protein were loaded per lane. Antibody dilutions were 1:100 for MID49 and 1:25,000 for GAPDH. Abbreviations: L, molecular weight ladder; WT, wild type; KO, MID49 knockout.

Supplementary Figure S9.

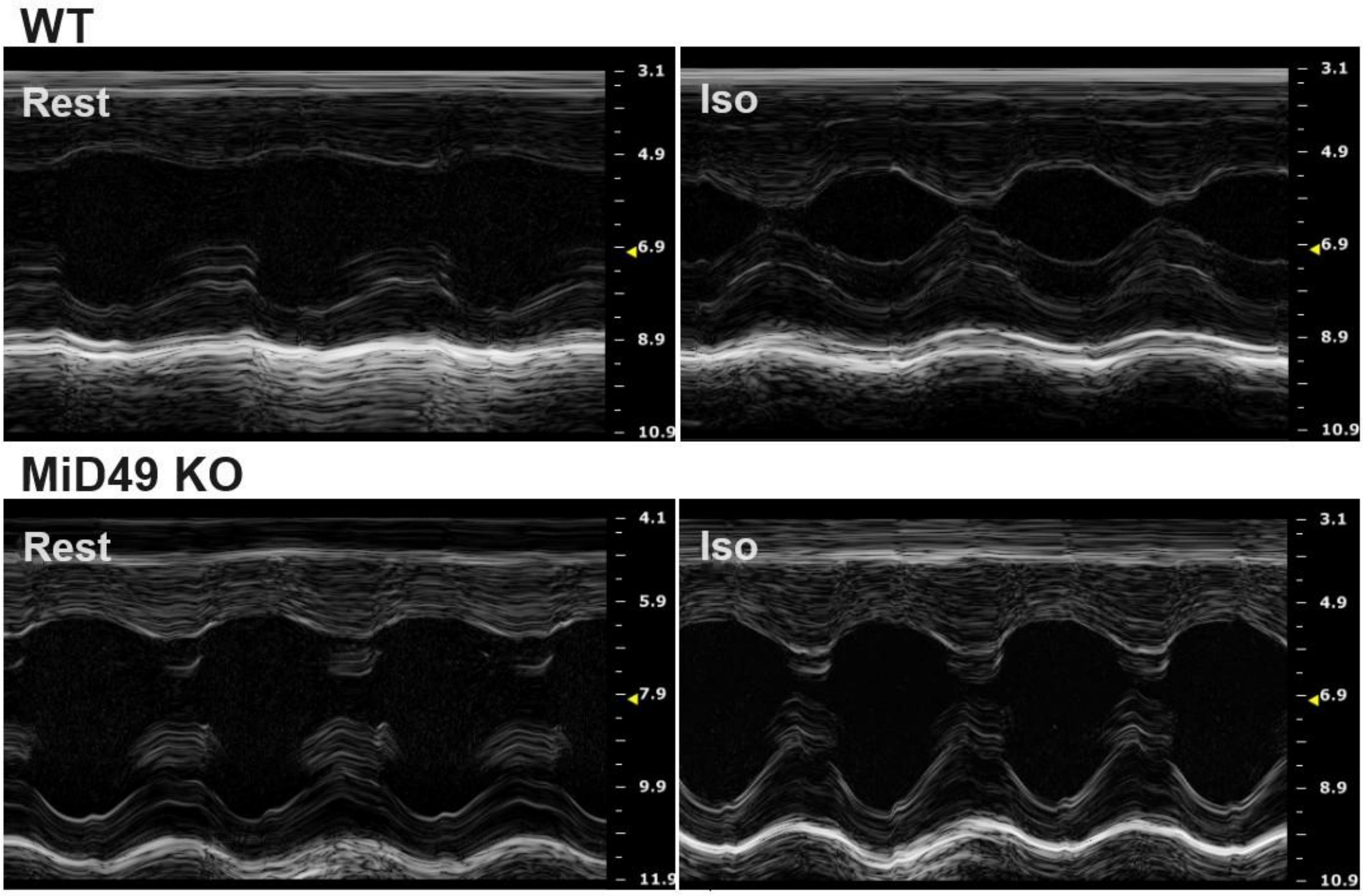

Figure S9. Enlarged echocardiographic images of wild-type and MiD49 knockout mice. Representative M-mode echocardiographic images, from Figure 5, in the parasternal long-axis (PLAX) view from wild-type (WT) and MiD49 knockout (KO) mice at rest and following intraperitoneal isoproterenol (Iso, 4 ng/g) administration.
