# Supplementary material for: Mitochondrial Dynamic Proteins MiD49 and MiD51 as Novel Targets of Cardioprotection
Source: Cells. 2026 Mar 20;15(6):559. doi: 10.3390/cells15060559 (PMC13024988; doi:10.3390/cells15060559)
Supplement: Supplementary file 1 [file cells-15-00559-s001.zip › Supplementary tables for MiD manuscript 2025.pdf]

Supplementary tables

Supplementary table S1.

**VC cells**		Hypoxia	Reoxygenation				
		120min	2min	5min	10min	15min	30min
Hypoxia	Basal	ns	***	***	**	**	**
	2min	ns	***	***	**	**	***
	20min	*	***	***	***	***	***
	40min	ns	***	***	**	**	**
	60min	ns	***	***	*	ns	*
	80min	ns	***	**	ns	ns	ns
	100min	ns	*	ns	ns	ns	ns
	120min		ns	ns	ns	ns	ns
Reoxygenation	2min			ns	ns	ns	ns
	5min				ns	ns	ns
	10min					ns	ns
	15min						ns

**MiD KD cells**		Hypoxia	Reoxygenation				
		120min	2min	5min	10min	15min	30min
Hypoxia	Basal	ns	*	***	ns	ns	ns
	2min	ns	***	***	ns	ns	ns
	20min	ns	***	***	ns	ns	ns
	40min	ns	***	***	ns	ns	ns
	60min	ns	***	***	ns	ns	ns
	80min	ns	***	***	ns	ns	ns
	100min	ns	***	***	ns	ns	ns
	120min		***	***	ns	ns	ns
Reoxygenation	2min			ns	ns	ns	ns
	5min				***	***	***
	10min					ns	ns
	15min						ns

Supplementary Table S1
Bonferroni's Multiple Comparison Test was used to identify significant changes in mitochondrial matrix calcium levels within VC and MiD KO cells, during RT SIRI, comparing individual timepoints within each condition.
